# Supplementary material for: The Mechanism of Manganese Ferrite Nanomaterials Promoting Drought Resistance in Rice
Source: Nanomaterials (Basel). 2023 Apr 26;13(9):1484. doi: 10.3390/nano13091484 (PMC10180523; doi:10.3390/nano13091484)
Supplement: Supplementary file 1 [file nanomaterials-13-01484-s001.zip › nanomaterials-2364800-supplementary.pdf]

Supplementary Materials

The Mechanism of Manganese Ferrite Nanomaterials Promoting Drought Resistance in Rice

Le Yue [1,2], Budiao Xie [1,2], Xuesong Cao [1,2], Feiran Chen [1,2], Chuanxi Wang [1,2], Zhenggao Xiao [1,2], Liya Jiao [1,2] and Zhenyu Wang [1,2,*]

[1] Institute of Environmental Processes and Pollution Control and School of Environment and Civil Engineering, Jiangnan University, Wuxi 214122, China;
[2] Jiangsu Engineering Laboratory for Biomass Energy and Carbon Reduction Technology, Wuxi 214122, China
* Correspondence: wang0628@jiangnan.edu.cn; Tel.: +86-0510-8591-1123

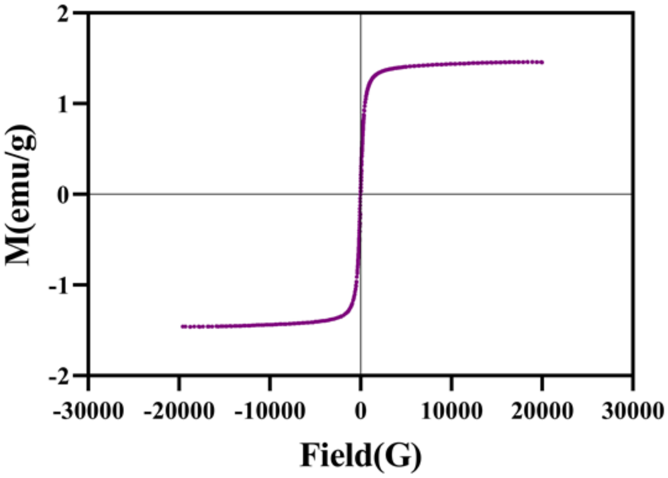

Figure S1. VSM curves of $MnFe_2O_4$ NMs.

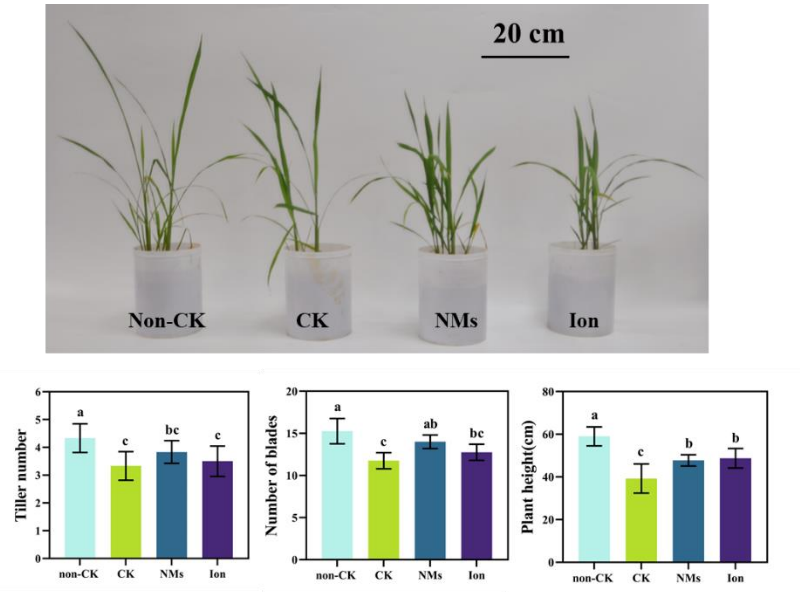

Figure S2. Rice growth as affected with 10 mg·kg^{-1} $MnFe_2O_4$ NMs and equivalent ion control under drought stress. The values are given as mean value ± standard deviation (n=5). Different letters mean significant differences ($p < 0.05$).

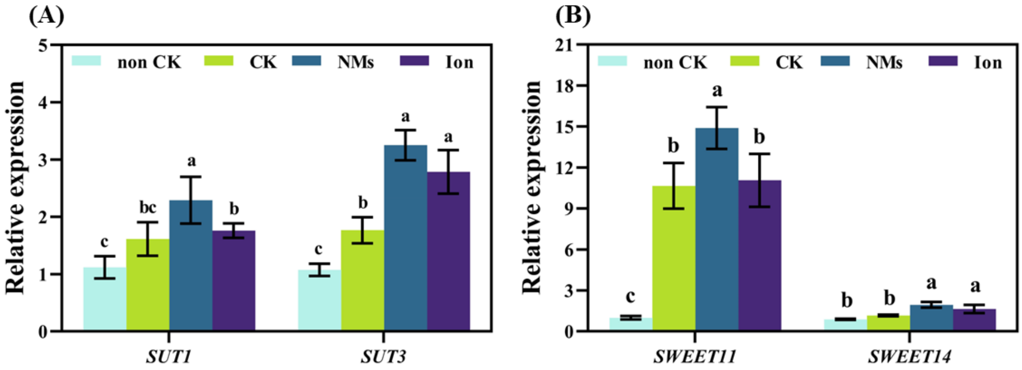

Figure S3. Relative expressions of SUT (A) and SWEET (B) in roots respond to 10 mg·kg^{-1} $MnFe_2O_4$ NMs and equivalent ion control under drought. The values are given as mean value ± standard deviation (n=5). Different letters mean significant differences ($p < 0.05$).

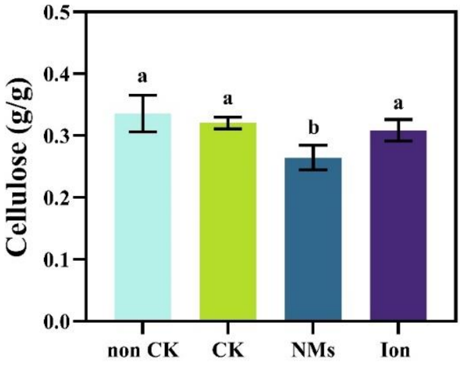

Figure S4. Cellulose content in rice roots responds to 10 mg·kg^{-1} $MnFe_2O_4$ NMs and equivalent ion control under drought. The values are given as mean value ± standard deviation (n=5). Different letters mean significant differences ($p < 0.05$).

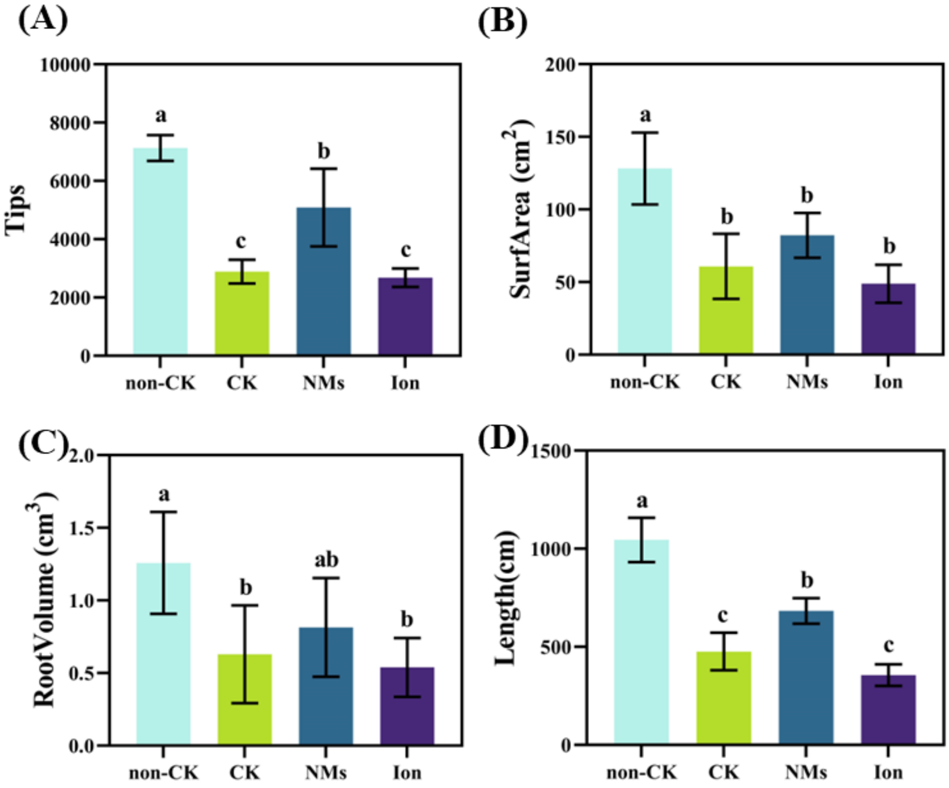

Figure S5. Root growth responds to 10 mg·kg^{-1} $MnFe_2O_4$ NMs and equivalent ion control under drought. (A) Root tip number, (B) root surface area, (C) root volume, and (D) root length. The values are given as mean value ± standard deviation (n=5). Different letters mean significant differences ($p < 0.05$).

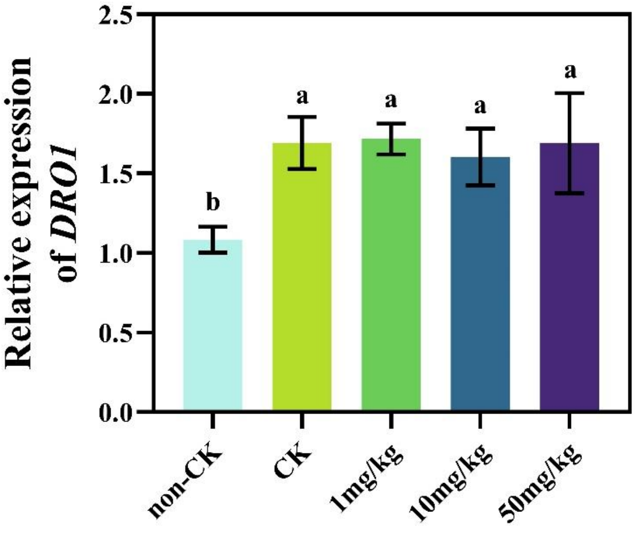

Figure S6. Relative expression of *DRO1* responds to 10 mg·kg^{-1} $MnFe_2O_4$ NMs and equivalent ion control under drought. The values are given as mean value ± standard deviation (n=5). Different letters mean significant differences ($p < 0.05$).

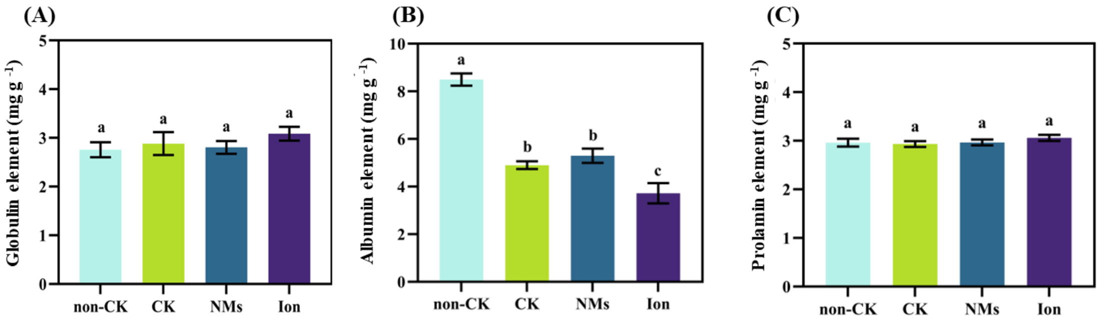

Figure S7. Globulins (A), albumin (B), and prolamin (C) contents respond to 10 mg·kg^{-1} $MnFe_2O_4$ NMs and equivalent ion control under drought. The values are given as mean value ± standard deviation (n=5). Different letters mean significant difference ($p < 0.05$).

Table S1. Primer set list.

Primer	Sequence (5’-3’)
AUX1-F	GCCTGCGCGAGTAACATCTA
AUX1-R	CAGCACCAGCTTGGTTGGAC
AUX2-F	GCCTGCGCGAGTAACATCTA
AUX2-R	CAGCACCAGCTTGGTTGGAC
AUX3-F	TTGGAATTTTGCAGGTGGCG
AUX3-R	ACCACTGGATGACGTGGTTC
PIN1a-F	TCATCTGGTCGCTCGTCTGC
PIN1a-R	CGAACGTCGCCACCTTGTTC
PIN2-F	CAGGGCTAGGAATGGCTATGT
PIN2-R	GCAAACACAAACGGGACAA
DRO1-F	GCAAGAAGCAAATCGGTTTCC
DRO1-R	GAATTCATCCTTTCGACAATCTGA
OR1-F	ACGATGCTTCACAAGAGGATGA
OR1-R	*CGGCTTATCCTCAAGATAGTATTTCC*
Ubi-F	AACCAGCTGAGGCCCAAGA
Ubi-R	ACGATTGATTTAACCAGTCCATGA
CLE25-F	GGTAAGGATGTGAATGTTCATGT
CLE25-R	TCTGCTTTCCTGTTGGGATAGG
NCED3-F	CACGATTCGCGATCAGAGAGA
NCED3-R	CCGGCAGCTTGAAACGA
SUT1-F	CATTCATCCTCTACGACACTGA
SUT1-R	CACAATCGAATTCAGTAGCAGG
SUT3-F	CTCGTTGAGTTAACTTCGTTGG
SUT3-R	TATTTACAGGGCCTGCCATAAA
SWEET11-F	AAGACCAAGAGCGTCGAGTT
SWEET11-R	ACGTTCGGGTACATGACGTA
SWEET14-F	TTCTTGGTTGGGTCTGCGTT
SWEET14-R	GAGGGAGAAGGAGAGGCGAGA

Table. S2 Zeta potential and hydraulic diameter of $MnFe_2O_4$ NMs.

	**$MnFe_2O_4$ NMs**
hydraulic diameter	982.0 ± 83.6nm
Zeta potential	-27.0 ± 1.2mV

Table S3. Contents of nutrient elements in rice after $MnFe_2O_4$ NM application.

	**treatments ($MnFe_2O_4$ NMs)**	**P**	**K**	**Ca**	**Mg**	**S**	**Fe**	**Mn**	**Cu**	**Zn**
	Non-CK	1096.5±68.4a	8714.3±789.8a	2745.3±362.0a	1968.8±129.8b	4769.5±1256.0ab	19388.7±987.3b	479.7±18.9a	30.5±3.5ab	19388.7±987.3b
	CK	590.1±26.9d	5740.7±125.0b	2709.1±355.2a	1780.4±134.7b	2616.7±451.3c	20203.4±948.0b	227.2±28.80b	24.5±3.02b	20203.4±948.02b
shoot	1 $mg \cdot kg^{-1}$	661.1±76.7cd	6030.8±510.9b	3369.9±1706.6a	1469.8±170.1b	3519.1±1226.4b	19026.0±2333.1b	221.8±41.9b	46.9±19.5ab	19026.0±2333.1b
	10 $mg \cdot kg^{-1}$	749.5±21.1b	5033.6±567.3b	2673.4±514.7a	1807.4±696.3b	3920.2±1875.5b	23426.7±2315.3a	348.6±208.2ab	47.8±20.5ab	23426.7±2315.3a
	50 $mg \cdot kg^{-1}$	639.2±70.2c	5513.3±949.6b	4992.5±2063.1a	2865.3±249.8a	5155.6±811.8a	5597.0±1585.8c	213.1±206.9b	61.0±26.0a	5597.0±1585.8c
	Non-CK	1871.5±197.1a	33501.3±3875.8b	5365.2±517.3a	2253.9±292.6bc	2561.7±128.3c	165.9±9.1a	273.2±61.9b	11.3±0.8c	165.9±9.1a
	CK	1478.7±82.8bc	32795.8±1784.5b	3518.4±699.4a	2085.1±62.0c	3052.1±174.4bc	128.8±9.9a	164.9±48.0b	12.2±0.5bc	128.8±9.9a
root	1 $mg \cdot kg^{-1}$	1408.4±75.3c	34237.9±2380.1b	5029.8±1506.8a	3032.3±461.2a	3124.0±204.5bc	163.8±23.1a	329.5±73.4b	11.6±2.5c	163.8±23.1a
	10 $mg \cdot kg^{-1}$	1687.8±106.7ab	41509.0±1071.6a	3471.7±1056.4a	3045.5±696.3a	3659.2±531.9ab	144.4±4.5a	242.1±72.6b	17.2±3.0a	144.4±4.5a
	50 $mg \cdot kg^{-1}$	1564.5±62.2bc	32043.4±4993.0b	5190.2±1700.1a	2739.8±232.9ab	3457.9±514.8ab	140.0±12.3a	644.3±239.8a	15.8±1.7 a	140.0±26.0a
